# Supplementary material for: Splice-Junction-Based Mapping of Alternative Isoforms in the Human Proteome
Source: Cell Rep. Author manuscript; Available in PMC 2020 Jan 15. (PMC6961840; doi:10.1016/j.celrep.2019.11.026)

A

sp|Q14997|PSME4_HUMAN|ENSG00000068878|SE1|33317|chr2|53940000|53942224|−1|r59|T1
YHSSVENILK q value: 0.00037993 Tr_novel:TRUE RefSeq_Novel:FALSE
Search result spec prec mz: 595.3162 Actual spec prec mz: 595.31617
Fragments matched per AA: 4.5 Proportion of top 20 peaks matched: 0.2

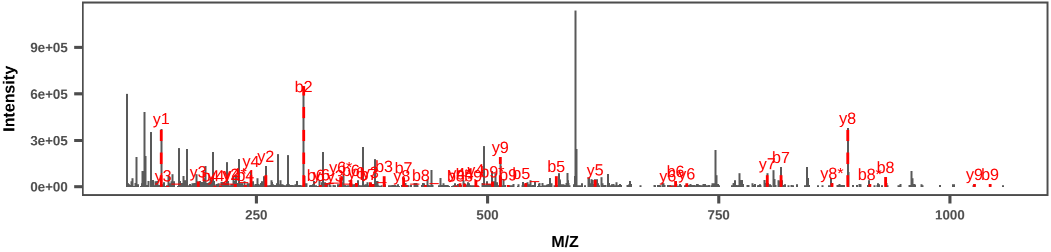

B

Scatterplot of predicted elution time
Fitting R2: 0.796
Novel peptide residual Z score: −0.836
Number of peptides: 4970

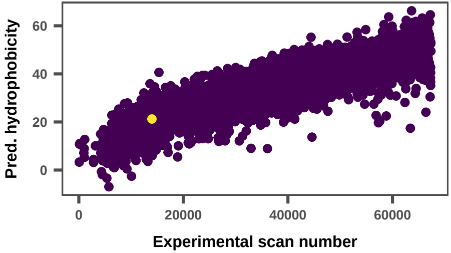

C

Distributions of residuals from best−fit line
of predicted RT vs Expt. scan number
Line: Z score of novel peptide
Z: −0.836

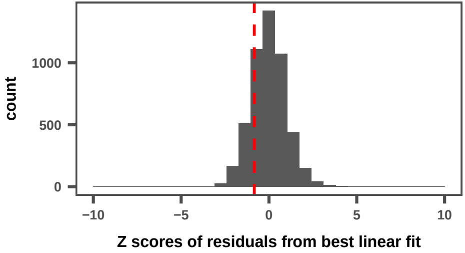

Supplement: 2 [file NIHMS1546469-supplement-2.zip › DF1/PXD009737/TestisTrypsin/TestisTrypsin_35_PSME4_YHSSVENILK.pdf]
